# Otolaryngology needs among an adult homeless population: a prospective study

**DOI:** 10.1186/s40463-020-00445-2

**Published:** 2020-07-09

**Authors:** Vincent Wu, Christopher W. Noel, David Forner, Florence Mok, Molly Zirkle, Antoine Eskander, Vincent Lin, John M. Lee

**Affiliations:** 1 Department of Otolaryngology, Head & Neck Surgery, University of Toronto, Toronto, Canada; 2 Department of Otolaryngology, Head & Neck Surgery, Dalhousie University, Halifax, Canada; 3 Department of Otolaryngology, Head & Neck Surgery, Sunnybrook Health Sciences Centre, Toronto, Canada; 4 Li Ka Shing Knowledge Institute, Toronto, Canada; 5 Department of Otolaryngology, Head & Neck Surgery, St. Michael’s Hospital, Toronto, Canada

**Keywords:** Homeless persons, Healthcare needs, Otolaryngology, Head and neck surgery, ENT

## Abstract

**Background:**

Homeless individuals frequently experience poor access to healthcare, delayed clinical presentation, and higher disease burden. Providing subspecialty otolaryngology care to this population can be challenging. We previously reported on the prevalence of hearing impairment in Toronto’s homeless community. As a secondary objective of this study, we sought to define otolaryngology specific need for this population.

**Methods:**

One hundred adult homeless individuals were recruited across ten homeless shelters in Toronto, Canada using a stratified random sampling technique. An audiometric evaluation and head and neck physical examination were performed by an audiologist and otolaryngology resident, respectively. Basic demographic and clinical information was captured through verbal administration of a survey. Descriptive statistics were used to estimate frequency of otolaryngology specific diseases for this population.

**Results:**

Of the 132 individuals who were initially approached to participant, 100 (76%) agreed. There were 64 males, with median age of 46 years (IQR 37–58 years). The median life duration of homelessness was 24 months (IQR 6–72 months). Participants had a wide range of medical comorbidities, with the most common being current tobacco smoking (67%), depression (36%), alcohol abuse (32%), and other substance abuse (32%). There were 22 patients with otolaryngology needs as demonstrated by one or more abnormal findings on head and neck examination. The most common finding was nasal fracture with significant nasal obstruction (6%). Eleven patients required referral to a staff otolaryngologist based on concerning or suspicious findings, including two head and neck masses, 6 were later seen in follow-up.

**Conclusion:**

There were substantial otolaryngology needs amongst a homeless population within a universal healthcare system. Future research should focus on further elucidating head and neck related issues in this population and expanding the role of the otolaryngologist in providing care to homeless individuals.

## Background

It is estimated that on any given night in Toronto, Canada, there are 5000 who are homeless. Over 28,000 different individuals access shelter services annually in the city [1, 2]. The homeless population encompasses some of the most vulnerable and underserved individuals in any community, experiencing substantial health inequities and disease burden [3, 4]. Recognizing that large national surveys target individuals living in private dwellings and inadvertently excluded vulnerable communities, significant effort has been made towards identifying the specific health needs of the homeless community. Literature has demonstrated that the homeless populations are susceptible to a wide range of medical comorbidities, including higher rates of diabetes, hypertension, cardiovascular disease, vision loss, hearing loss, chronic obstructive pulmonary disease, and human immunodeficiency virus infections [3–10].

Additionally, homeless individuals experience poor access to healthcare, driven by factors that include lack of health insurance coverage, prioritization of other needs (i.e. food and shelter), and previous experiences that were negative or discriminatory within the healthcare system [11]. Together, these circumstances often translate into delayed clinical presentation of diseases, increased rates of hospitalization often for preventable conditions, and higher rates of mortality [12]. Within the surgical literature, one study found only half of referred patients who were homeless attended outpatient appointments, with only one third completing full follow up – approximately half the rate as compared to the general public [13]. Similarly, previous experiences of discrimination in the healthcare system, lack of financial resources, high transportation costs, and absence of insurance coverage were identified as barriers for seeking surgical care in the homeless [13].

In an attempt to better understand the otolaryngology needs in the homeless population, our group previously evaluated rates of uncorrected hearing loss amongst adults within homeless shelters in Toronto. We identified significantly higher rates of speech-frequency hearing loss and high-frequency hearing loss in the homeless as compared to the general Canadian population [10]. As a secondary objective, we also provided screening for all participants for any disease processes typically treated by otolaryngologist. To the authors’ knowledge, only one study had previously detailed otolaryngology specific needs in this vulnerable population. Moore et al. noted that 41% had an otolaryngology complaint [14]. Results were drawn from a United States population, through a convenience sample, and may not be applicable to homeless individuals living within a system of universal health insurance, such as Canada. We build upon these results by reporting results of otolaryngology specific needs from a stratified random sample of an urban adult homeless population in a large Canadian city.

## Methods

This was a prospective cross-sectional study. We defined “homelessness” as any person residing in a homeless shelter for a minimum of 7 consecutive days [10]. Only English speaking participants with decisional capacity were eligible for inclusion [15].

### Participant selection

A randomized two-stage sampling technique was employed between April and June 2018, in recruiting participants from adult homeless shelters in the city of Toronto, Canada. All adult homeless shelters with 20 or more beds were identified. Ten shelters were randomly selected from this list, with the probability of selection proportional to each shelter’s housing capacity. Simple randomization via a random number generator (www.random.org) was then used to select bed numbers. Individuals assigned to the randomly selected beds were invited to participate in the study. This process continued until 10 participants had been recruited from each shelter, for a total of 100 participants. This was a pilot study designed to estimate prevalence of hearing impairment within a homeless population, and as a result, a sample size was not calculated. Participants were excluded if they were less than 18 years of age, non-English speakers, or lacked decisional capacity.

### Survey

All interviews were conducted by CWN using a modified data sheet used in prior studies to assess health needs of homeless populations [10]. The survey included questions on participant demographics, past medical history, access to healthcare/medical devices, noise exposure, subjective measure of hearing, and a hearing handicap screening questionnaire. Participants who had indicated “smoking” or “alcohol use” within the medical history section of the survey were asked specifically about symptoms of head and neck cancer, along with their understanding of head and neck cancer, and its associated symptoms. Participants received a $10 gift card after completion of the study.

### Head and neck assessment

Head and neck examination was performed by an otolaryngology resident physician (CWN) on all participants at the shelter. This included pneumatic otoscopy and a thorough examination of each individual’s head and neck including the nasal cavity, oral cavity, oropharynx, and neck. Other otolaryngology needs arose during the physical examination, and additional open-ended questions were employed as appropriate to determine participant symptoms (i.e. obvious nasal fracture with deviated nasal septum, whether participant is experiencing nasal obstruction). In the event that the otolaryngology resident carried a high degree of clinical suspicion for a nasopharyngeal or laryngeal pathology, examination with a flexible endoscope was performed. Findings of suspicion and/or concern were referred on to a staff otolaryngologist (MZ) for further assessment in hospital.

### Outcome measures

Demographic information was collected for each participant. Primary outcome measures included rates of otolaryngology needs, and positive findings of pathology or concern on the head and neck examination. Secondary outcome measures included co-morbidities and previous assessment/surgery by an otolaryngologist.

### Statistical analysis

Data was imported into a spreadsheet program (Excel, Microsoft, United States) designed a priori for the study. Descriptive statistics including median and inter-quartile range (IQR), as well as mean and standard deviation (SD), were used in displaying the data as appropriate following an assessment of normality. Categorical variables were reported as frequencies and relative frequencies.

## Results

Of the 132 homeless individuals initially approached to participant in the study, 100 (76%) agreed. As someone within the patients inner of circle of care made the initial approach, we were unable to identify reasons for refusal. There were 64 male participants, with median age of 46 years (IQR 37–58 years). The median life duration of homelessness was 24 months (IQR 6–72 months). Most participants were high school graduates (91%) and 35% had some post-secondary education. The majority of participants were living on less than $500 Canadian dollars per month. Participants’ demographic information are outlined as part of Table 1. All participants were eligible for the Ontario Health Insurance Plan (OHIP) and most participants (78%) had some form of extended health care benefits through social assistance that covers the cost of medication, medical assisted devises and other services not covered through OHIP (either Ontario Works or the Ontario Disability Support Program).

**Table 1:** Demographic characteristics of participants

Characteristic	***n*** = 100
Sex	
Male	64
Female	36
Age, median 46 years (IQR 37–58 years)	
18–29	8
30–39	24
40–49	22
50–59	26
60+	20
Length of Time Spent Homeless, median 24 months (IQR 6–72 months)	
< 1 year	38
1–5 years	33
> 5 years	29
Ethnicity	
White	57
Black	35
Indigenous/Aboriginal	4
Asian	4
Marital Status	
Single	66
Married or Common-law	9
Divorced, Separated, or Widowed	24
Refused	1
Highest Level of Education Achieved	
Elementary School	1
Middle School	8
High School	56
College/University	35
Monthly Income, $ (Canadian dollars)	
<$500	45
$500–$1000	23
>$1000	22
Refused	10

Participants had a wide range of medical comorbidities, with the most common self-reported health issues being active tobacco smoking (67%), depression (36%), alcohol abuse (32%), other substance abuse (32%), hypertension (22%), and asthma (19%). The average pack year history of tobacco smoking was 26 years (SD 22 years). Eight participants recalled having been assessed and/or treated by an otolaryngologist in the past. Previous surgical procedures included tonsillectomy, myringotomy and tympanostomy tube insertion, tympanoplasty, translabyrinthine resection of a meningioma, functional rhinoplasty, and incision and drainage of deep neck space infection.

**Fig. 1:**
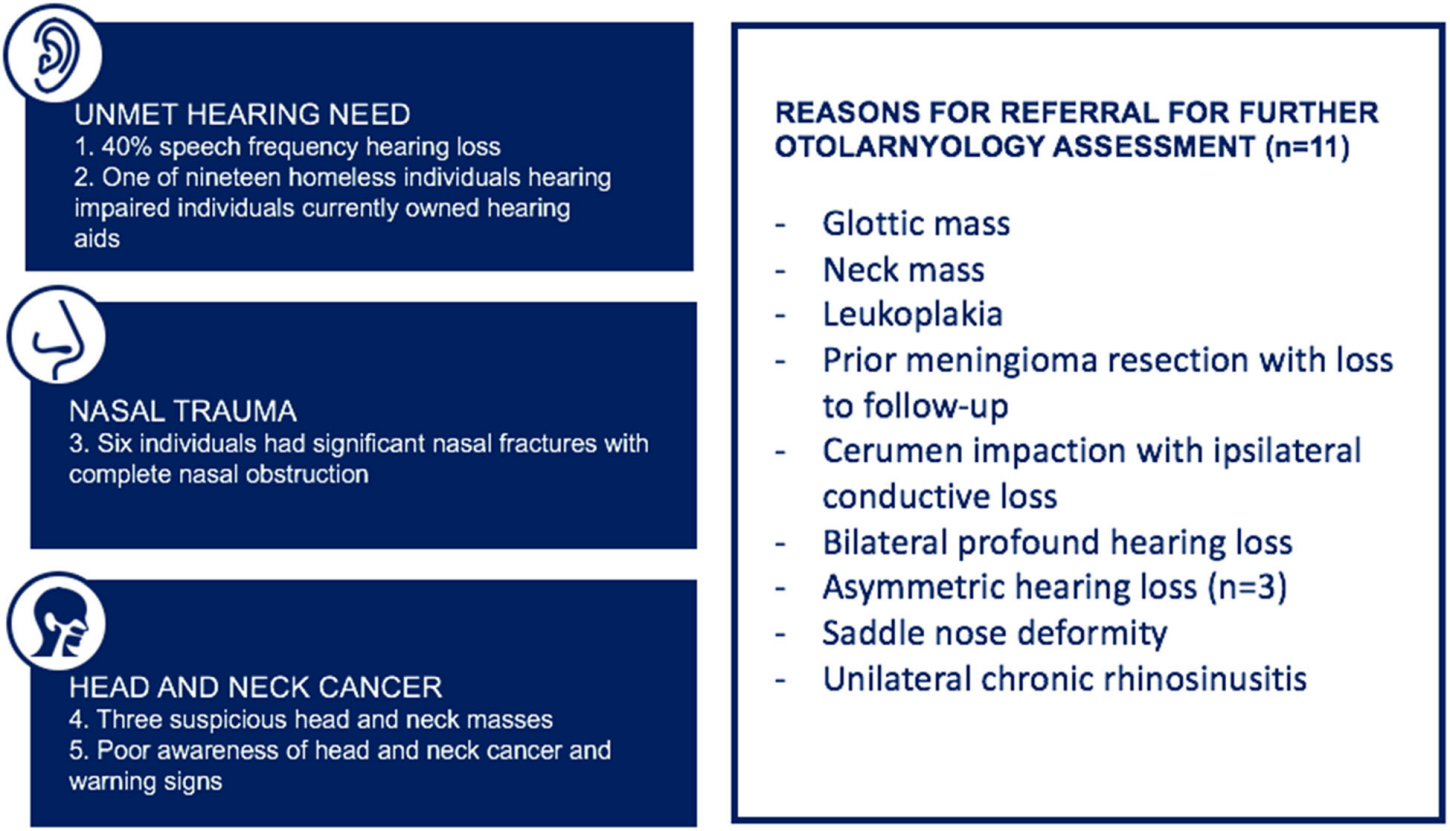
Results from assessment of randomly selected homeless individuals

In total, there were 22 patients who had one or more abnormal findings on the head and neck examination. The most common abnormal finding was nasal fracture with associated significant nasal obstruction (6%), followed by poor dentition (3%). Nasal fractures were all longstanding fractures with history of polytrauma. A number of patients did not want to be referred and/or refused further medical intervention. Ultimately, eleven patients were referred to a staff otolaryngologist based on abnormal findings on physical exam. The indications for referral included suspected malignancy (glottic mass, neck mass, oral leukoplakia), prior meningioma resection with loss to follow-up, cerumen impaction with ipsilateral conductive loss, bilateral profound hearing loss, asymmetric hearing loss (*n* = 3), saddle nose deformity, and unilateral chronic rhinosinusitis (Fig. 1). Six of these 11 indiviudals eventually were seen in follow-up (5 by an otolaryngologist, 1 by a general practitioner). In an effort to retain annonymity and patient confidentiality, we are unable to discuss individual trajectories of each participant.

## Discussion

Otolaryngology needs in the homeless were assessed in this study, with 22% having one or more abnormal findings and 11% requiring referral for specialist assessment. We found several diseases among participants, including possible head and neck malignancies. The most frequently noted abnormal finding was nasal fracture associated with nasal obstruction amongst participants in our study. An 8-year retrospective study in Ontario, Canada found that, among selected conditions, facial fractures represented the third most common reason for presentation to the emergency department among the general public, most likely as a result of falls, being struck by a person and/or object, and traffic collisions [16]. The estimated rate of facial trauma presentation to the emergency department was 967 per 100,000 [16]. Given the association of facial/nasal fractures with trauma, and increased susceptibility of homeless individuals to traumatic injuries, it is not surprising that nasal fractures are frequently encountered in this population [17].

It is widely known that head and neck cancers are associated with exposure to alcohol and tobacco, which are both associated with advanced tumors at presentation and increased hazard for death [18]. Exposure to tobacco and alcohol were the first and third most commonly reported health comorbidities in our study population, respectively. Westerberg et al. reviewed otolaryngology-related disorders in underserved and vulnerable populations and noted various social determinants within this population have traditionally contributed to delays in seeking medical attention, which may lead to advanced stage of disease at presentation [19].

The authors previously published a pilot study, evaluating hearing needs in homeless individuals, and found significantly higher rates of speech-frequency hearing loss (39.5%, [95% confidence interval (CI) 30.4–49.3%]) and high-frequency hearing loss (51.9%, [95%CI 42.2–61.4%]) in the homeless population as compared to the general Canadian public (speech-frequency: 19.2% [95% CI 16.9–21.7%] and high-frequency: 35.5% [95%CI 33.1–37.7%]) [10]. In this submission, we reported on healthcare needs that would benefit from otolaryngology assessment. In comparing our results to the study by Moore et al., who assessed rates of undiagnosed head and neck malignancies in homeless adults, the proportion of otolaryngology needs was comparable. In their study, convenience sample was used to recruit 235 homeless individuals in the United States. Moore et al. reported 41% of the participants had an otolaryngology complaint, and 11% were subsequently referred on for head and neck cancer evaluation. Of the referred patients, 80% underwent further testing including biopsy, with two individuals receiving treatment for head and neck malignancies [14]. However, with different sampling methodologies, even as we employed a random sampling technique, we were still able to capture a high rate of abnormal findings on physical exam, thereby affirming the high degree of otolaryngology needs in this population. Interviewer observation also suggests a substantial proportion of the sample population and shelter staff were unaware of the association between smoking, alcohol use, and head and neck cancer. As this was not a primary or secondary objective of the study, data was not formally recorded. However, this highlights an important issue for future research, patient advocacy, and healthcare promotion within this population.

Within the general public, barriers in accessing specialist care have been identified. Fradgley et al. found through their systematic review of the literature that the most common barriers encountered by patients in accessing specialist care included transportation costs, scheduling conflicts, poor communication, and lack of coordination within the healthcare system [20]. Specific barriers that have been identified across homeless individuals include previous negative experiences with the healthcare system or previous discrimination within a healthcare setting, lack of medical insurance, and prioritization of other needs (i.e. food and shelter) above their health [11]. In a recent study by Zuccaro et al. evaluating surgical care needs of homeless patients in Ottawa, Canada, follow up rates were reported to be approximately one half of referred patients [13]. Within our studies, patients with findings that were suspicious or concerning were referred for assessment by a staff otolaryngologist in an outpatient clinic within a tertiary hospital setting. In organizing in-hospital specialist follow up, we arranged for paid transportation to and from the shelter for all referred participants. Of the eleven who were referred, only half completed follow up, which was similar to the findings reported by Zuccaro et al. Unfortunately, due to confidentiality restraints, reasons for missed appointments were unable to be evaluated in this study. Ensuring ongoing care is provided in this patient group remains an important aspect to address.

The results of this study must be interpreted in the context of its cross-sectional design, as a purely descriptive study. Firstly, although the Toronto shelter system is known to represent the majority of the homeless population, there is still a significant cohort of individuals who do not access shelter service, who may have been missed as a result of our sampling strategy [21]. Much of our survey results relied on participant recall and are subsequently prone to bias. There was also the potential for non-respondent bias given that 25% of individuals approached declined participation in the study. Not all participants had flexible nasopharyngoscopy performed; only individuals who were highly suspected of either a nasopharyngeal or laryngeal pathology had an endoscopic evaluation performed. Ideally, a complete examination with endoscopy would be performed, given that this population is at inherently higher risk of head and neck cancer, but was limited by portable equipment and resources available to be utilized while visiting the homeless shelters. Studies in the future can aim to identify barriers to access, formulating ways of increasing specialty care for homeless individuals. Finally, without a matched comparator group, we are unable to infer causal inference.

Other centers across Canada, including the University of Ottawa [22] and the University of British Columbia [23], have established outreach programs, aimed at head and neck cancer screen in the homeless populations and other vulnerable populations. Our group has been sponsored by the American Head and Neck Society through a grant in order to conduct head and neck cancer screening in adult homeless shelters across Toronto, to coincide with head and neck cancer awareness week. We believe that bringing care to the homeless, in spaces and places that they are familiar and comfortable with, can help breakdown some of the systematic and social barriers which imped their access to appropriate healthcare [11, 13, 19].

## Conclusion

We found that 22 (22%) participants had abnormal head and neck exam findings, of which 11 were ultimately referred on to a staff otolaryngologist. Homeless individuals have high rates of medical comorbidities commonly associated with head and neck malignancies even when living in systems of universal health insurance. The authors believe that future outreach and screening programs can help to identify and address inequities in otolaryngology disease in the homeless population.

## Data Availability

The datasets used and/or analyzed during the current study are available from the corresponding author on reasonable request.

## Abbreviation

CI: Confidence interval
IQR: Inter-quartile range
SD: Standard deviation

## References

[1] City of Toronto Shelter Support and Housing Administration (2010). Per Diem Rates for the Purchase of Service Shelter System and Results of the Review of the Per Diem Funding Model.

[2] City of Toronto Shelter Support and Housing Administration (2006). Street Needs Assessment results. 2006 TCoT.

[3] Hwang SW (2001). Homelessness and health. CMAJ..

[4] Hwang SW, Bugeja AL (2000). Barriers to appropriate diabetes management among homeless people in Toronto. CMAJ..

[5] Gelberg L, Linn LS (1989). Assessing the physical health of homeless adults. JAMA..

[6] Kushel MB, Vittinghoff E, Haas JS (2001). Factors associated with the health care utilization of homeless persons. JAMA..

[7] Jiang S, Mikhail M, Slomovic J, et al. Prevalence and impact of eye disease in an urban homeless and marginally housed population. Can J Ophthalmol. 2020;55(1):76–81. 10.1016/j.jcjo.2019.07.006 31712023

[8] Noel CW, Fung H, Srivastava R (2015). Visual impairment and unmet eye care needs among homeless adults in a Canadian city. JAMA Ophthalmol.

[9] Noel CW, Srivastava R, Lo R, Berger A, Tehrani N, Lichter M (2016). Unmet eye care needs among a homeless youth population. Can J Ophthalmol.

[10] Noel CWMF, Wu V, Eskander A, Yao C, Hwang SW, Lichter M, Reekie M, Smith S, Syrett I, Zirkle M, Lin V, Lee JM (2020). Hearing loss and hearing needs in an adult homeless population: a prospective cross-sectional study. Can Med Assoc J Open.

[11] Khandor E, Mason K, Chambers C, Rossiter K, Cowan L, Hwang SW (2011). Access to primary health care among homeless adults in Toronto, Canada: results from the street health survey. Open Medicine.

[12] Baggett TP, O'Connell JJ, Singer DE, Rigotti NA (2010). The unmet health care needs of homeless adults: a national study. Am J Public Health.

[13] Zuccaro L, Champion C, Bennett S, Ying Y (2018). Understanding the surgical care needs and use of outpatient surgical care services among homeless patients at the Ottawa Hospital. Can J Surg.

[14] Moore CE, Durden F (2010). Head and neck Cancer screening in homeless communities: HEAL health education, assessment, and leadership. J Natl Med Assoc.

[15] Etchells E. Aid to capacity evaluation (ACE) Toronto: Joint Centre for Bioethics; 2015.

[16] Al-Dajani M, Quiñonez C, Macpherson AK, Clokie C, Azarpazhooh A (2015). Epidemiology of maxillofacial injuries in Ontario, Canada. J Oral Maxillofac Surg.

[17] Mackelprang JL, Graves JM, Rivara FP (2014). Homeless in America: injuries treated in US emergency departments, 2007–2011. Int J Inj Control Saf Promot.

[18] Adoga AA, Kokong DD, Ma’an ND, Mugu JG, Mgbachi CJ, Dauda AM (2018). The predictive factors of primary head and neck cancer stage at presentation and survival in a developing nation’s tertiary hospital. SAGE Open Med.

[19] Westerberg BD, Lango MN (2018). Otolaryngology-related disorders in underserved populations, otolaryngology training and workforce considerations in North America. Otolaryngol Clin N Am.

[20] Fradgley EA, Paul CL, Bryant J (2015). A systematic review of barriers to optimal outpatient specialist services for individuals with prevalent chronic diseases: what are the unique and common barriers experienced by patients in high income countries?. Int J Equity Health.

[21] Hwang SW, Martin RE, Tolomiczenko GS, Hulchanski JD (2003). The relationship between housing conditions and health status of rooming house residents in Toronto. Can J Public Health.

[22] Global Health/Outreach. Department of Otolaryngology – Head and Neck Surgery University of Ottawa. https://med.uottawa.ca/otolaryngology/about/global-healthoutreach. Accessed March 11, 2020.

[23] St. Paul’s Hospital. St. Paul’s otolaryngologists establish cancer screening clinic in the DTES Web site. https://helpstpauls.com/2016/12/06/st-pauls-otolaryngologists-establish-cancer-screening-clinic-dtes Accessed March 11, 2020.

